# A Comparative Study of Oral Health and Behavior Among 7–9-Year-Old Iranian and Afghan Children

**DOI:** 10.31661/gmj.v13iSP1.3754

**Published:** 2024-12-29

**Authors:** Mohammad Rahimi Jafari, Mojgan Faezi, Seyed Masoud Sajedi

**Affiliations:** ^1^ Dentist, Varamin Health Centre, Varamin, Iran; ^2^ Department of Community Oral Health, Faculty of Dentistry, Shahed University, Tehran, Iran; ^3^ Department of Oral and Maxillofacial Medicine, Faculty of Dentistry, Shahed University, Tehran, Iran

**Keywords:** Dental Caries, Dmft Index, Pediatric Dentistry, Afghan Immigrants, Oral Health Disparities, Dental Public Health, Health Behavior, Iran

## Abstract

**Background:**

Afghan immigrants represent the largest foreign-born population residing in Iran. the present study aimed to perform a comparative evaluation of the dmft index and oral health behaviors among Iranian and Afghan children aged 7 to 9 years residing in Pishva County.

**Materials and Methods:**

In this 2023 cross-sectional study, 157 children (87 Iranian and 70 Afghan) were recruited via convenience sampling. Participants were selected from elementary schools in Pishva County, Iran. Oral health behaviors were evaluated using the culturally adapted Oral Health Behavior Self-Reporting Questionnaire Parents completed these self-administered questionnaires designed to capture information on their children’s oral health behavior, source of information and school grade level. Statistical analyses were performed using SPSS 21 statistical software through Chi-square, Shapiro-Wilk test, General Linear Model (GLM), Mann Whitney U and Fisher’s exact tests.

**Results:**

A comparison of dmft categories revealed that Iranian children had a lower prevalence of severe dental caries: 32% were classified as "High" and 3% as "Very High," whereas 39% of Afghan children were classified as "High" and 27% as "Very High" (P0.001). Oral Health Behavior scores were significantly higher in the Iranian cohort (mean ± SD: 6.70 ± 1.37) compared to the Afghan cohort (mean ± SD: 4.50 ± 1.43; P 0.001). In the GLM predicting the dmft index, ethnicity emerged as a significant independent predictor (Wald χ²₁ = 70.806, P = 0.005). Additionally, the three-way interaction among ethnicity, school grade level, and oral health behavior was highly significant (Wald χ²₅ = 22.472, P 0.001). However, the main effects of school grade level and oral health behavior, when considered independently, did not reach statistical significance. It was observed that younger children had higher dmft scores, suggesting that age is a contributing factor in the prevalence of dental caries. No statistically significant difference was observed between the groups in terms of gender (P = 0.078). However, a significant difference was found in school grade level between the two groups (P = 0.018), as indicated by the chi-square test.

**Conclusion:**

The findings indicate that Iranian children aged 7–9 years generally demonstrate better oral health status and more favorable oral hygiene behaviors compared to Afghan immigrant children. Afghan children had both lower levels of oral health performance compared to Iranian children. These observed disparities underscore the influence of migration-related determinants on pediatric oral health, highlighting the necessity for culturally sensitive public health interventions targeting immigrant populations.

## Introduction

The long-standing of Afghan migration to Iran has culminated in the formation of the country’s largest immigrant community, with a substantial proportion now comprising second- and third-generation individuals born, raised, and educated within Iran. Common linguistic, religious, and cultural ties have facilitated the gradual process of integration. According to the Iranian Ministry of Foreign Affairs, by 2020, approximately 839,912 Afghan refugees held legal residency, an estimated 450,000 possessed temporary passports, and around 734,622 individuals lived without formal documentation [1].

Growing evidence suggests that immigrant children face disproportionately higher unmet oral health needs and are at elevated risk for dental pathologies compared to native-born peers [2]. Contributing factors include socioeconomic disadvantages, restricted access to dental care services, and systemic obstacles to obtaining health insurance [3][4][5]. Furthermore, many Afghan immigrants originate from regions where healthcare infrastructures are underdeveloped, and preventive oral health practices are minimally emphasized [6].

Despite notable declines in the prevalence of pediatric dental caries in many high-income settings, dental caries remains the most common chronic disease among children worldwide, particularly in low- and middle-income contexts. The pathogenesis of caries is influenced by biological susceptibility, behavioral factors, socioeconomic status, and cultural practices [7]. If left untreated, early childhood caries can lead to significant health consequences, including dental infections, impaired masticatory function, and reduced quality of life [8].

The Decayed, Missing, and Filled Teeth (DMFT/dmft) index serves as an internationally accepted epidemiological tool for assessing oral health status, particularly during the mixed dentition phase that typically occurs between ages 7 and 9 [9]. During this developmental stage, parental influences play a decisive role in shaping children’s oral hygiene behaviors [7].

Iran currently hosts approximately 1.58 million Afghan immigrants, with notable concentrations in Tehran Province, including Pishva County, where Afghan nationals represent a significant portion of the population [10]. In Pishva, Afghan immigrants are predominantly employed in labor-intensive sectors such as agriculture, construction, and animal husbandry [11].

Although documented Afghan immigrants have formal access to healthcare services, persistent disparities in oral health outcomes point to deeper sociocultural and structural inequalities. Accordingly, the present study sought to comparatively assess the dmft index and oral health behaviors among Iranian and Afghan children living in Pishva County, with the ultimate goal of informing the design of culturally attuned public health interventions [12].

## Materials and Methods

### Study Design and Setting

A cross-sectional study was conducted in 2023, involving 157 children aged 7 to 9 years, including both Iranian and Afghan nationals residing in Pishva County, Iran. The study protocol strictly complied with the ethical principles outlined in the Declaration of Helsinki of the World Medical Association [13]. Prior to enrollment, comprehensive information about the study’s aims and procedures was provided to parents or legal guardians to facilitate informed consent. Ethical approval for the study was granted by the Ethics Committee of Shahed University on May 8, 2023 (Approval ID: IR.SHAHED.REC.1402.020).

### Eligibility Criteria

Children were eligible for inclusion if they met the following criteria: (i) Iranian or Afghan nationality; (ii) aged between 7 and 9 years; (iii) current residence in Pishva County; and (iv) for Afghan participants, registration of birth in Iran and a documented family history of continuous residency for at least ten years [14]. Children demonstrating significant dental anxiety or declining participation were respectfully excluded.

### Sample Size Determination

The sample size was calculated using G*Power software (version 3.1.9.6), with parameters set to detect an effect size of 0.47, assuming a mean difference of 0.9 in dmft scores and a standard deviation of 1.9. Considering a significance level (α) of 0.05 and a power of 80%, a minimum of 157 participants was deemed necessary, comprising 70 Afghan and 87 Iranian children.

### Data Collection Procedures

Ethical approval for the study was obtained from the Ethics Committee of Shahed University on May 8, 2023 (Approval ID: IR.SHAHED.REC.1402.020). After coordination with the Ministry of Education and school administrators, three elementary schools—one rural, one boys’ school, and one girls’ school—were selected using convenience sampling. These schools were chosen based on practical considerations such as administrative feasibility and cooperation of school authorities. They were also strategically located across urban, suburban, and rural areas of Pishva County to enhance geographic and demographic diversity. According to data from the local Department of Education, the socioeconomic and demographic characteristics of enrolled students closely matched the broader school-age population in the region. Although convenience sampling involves a risk of selection bias, efforts were made to improve representativeness. The authors acknowledge this limitation and suggest that future research use randomized sampling methods to improve generalizability.

Clinical dental examinations were performed under optimal lighting using a dental explorer and mirror, following the World Health Organization (WHO) guidelines for oral health assessment [6]. The number of decayed (d), missing (m), and filled (f) primary teeth was recorded, and used to calculate the dmft index. The dmft index categories were defined as follows:

• Very low (<1.2)

• Low (1.2-2.6)

• Moderate (2.7-4.4)

• High (4.5-6.4)

• Very high (≥6.5)

In epidemiological studies of oral health, the DMFT and dmft indices are widely used to assess dental caries experience. The DMFT index measures the number of decayed, missing, and filled permanent teeth, whereas the dmft index refers to the same parameters but in primary (deciduous) teeth. The use of uppercase (DMFT) or lowercase (dmft) letters distinguishes between permanent and primary dentitions, respectively. These indices provide essential insights into the burden of dental caries across different age groups and are fundamental tools in international oral health surveys and research initiatives [7].

In addition to clinical assessments, oral health behaviors were evaluated using the culturally adapted Oral Health Behavior Self-Reporting Questionnaire [15], originally demonstrating a content validity of 77% and a Cronbach’s alpha coefficient of 88%. Following adaptation for cultural relevance within the Iranian context, three items were removed—one assessing English language proficiency, which was deemed unnecessary for this study, and two concerning income level and work schedule, as families declined to provide precise information—yielding a final 19-item questionnaire with a content validity index of 80% and a reliability score of 90%, respectively.

Due to the reluctance of Afghan families to provide accurate information regarding parental education levels, insurance possession and occupational status, these variables were excluded from our analysis. This decision was made to minimize the potential of confounding effects that unverified data could introduce into our study.

The oral health behavior questionnaire consisted of 14 items. For each block of behavior questionnaire, correct answers were chosen by the investigators. Each item was scored using one for a correct answer and zero for an incorrect answer. Subjects who had chosen ‘do not know’ item or left items blank were given a score of zero for that item. Since there was14 questions about dental behavior, the maximum score possible was 14. Relevant statistical tests were used confirming strength and validity of the results in connection to our study objective. In this section we first used Fisher’s exact test and Mann Whitney u test following to it.

Source of oral health information variable obtained included parents/ relatives, television and others (dentists/ health centers).

Demographic variables collected included gender, school grade level, ethnicity and the family’s length of residence in Iran.

## Statistical Analysis

Data analysis was performed using SPSS version 21. Fisher’s exact test was employed to analyze qualitative binary variables, ensuring the robustness and validity of the results in alignment with the study’s objectives. To assess the normality of the distribution of quantitative variables, the Shapiro-Wilk test was applied, allowing for the appropriate selection of statistical tests. This procedure facilitated the determination of whether the data followed a normal distribution, thus guiding the choice between parametric or non-parametric tests.

The Chi-square test was used to examine the relationship between categorical variables with three or more levels, assessing statistical independence or association between them. For the analysis of non-parametric qualitative binary variables, the Mann-Whitney U test was applied, further reinforcing the reliability of the findings in relation to the study’s aims.

To analyze the relationship between the dependent variable, the decayed, missing, and filled teeth index (dmft), and the independent variables—school grade level (SGL), ethnic group (categorical ethnic grouping), and oral health behaviors (OHB)—the Generalized Linear Model (GLM) with a Poisson distribution and a natural logarithm link function was utilized.

## Results

**Table:** Table1. Demographic Information of Participants

Variable	Iranian (n=87)	Afghan (n=70)	P-value	Test Type
Gender - Male	52 (59.8%)	33 (47.1%)	0.078	Fisher's Exact Test
Gender - Female	35 (40.2%)	37 (52.9%)		
Grade - First	47 (54.0%)	25 (35.7%)	0.018	Chi-Square Test
Grade - Second	25 (28.7%)	20 (28.6%)		
Grade - Third	15 (17.2%)	25 (35.7%)		

**Table:** Table2. Dental Health Status (dmft) of Children by Grade level

Grade	Very Low (%)	Low (%)	Moderate (%)	High (%)	Very High (%)
First	1%	4%	11%	20%	9%
Second	0%	1%	16%	10%	3%
Third	1%	3%	14%	5%	3%

**Table:** Table3. Dental Health Status (dmft) by Ethnic Group

Ethnicity	dmft Score	Frequency	Percentage (Per group)	P-value
	Very low	1	1%	
	Low	7	8%	
Iranian	Moderate	48	55%	
	High	28	32%	
	Very high	3	3%	< 0.001
	Very low	2	3%	
	Low	5	7%	
Afghan	Moderate	17	24%	
	High	27	39%	
	Very high	19	27%	

**Table:** Table4. Comparison of Oral Health Behaviors of Students Aged 7-9 in Pishva County by Ethnicity

Variable	Correct Answer	Iranian (n = 87)	Afghan (n = 70)	P-value
Purpose of toothbrushing	To prevent tooth decay	52 (59.8%)	30 (42.9%)	0.026*
Best type of toothbrush	Soft-bristled	22 (73.3%)	8 (26.7%)	0.022*
Toothbrush replacement frequency	Every three months	45 (80.4%)	11 (19.6%)	<0.001*
History of toothache	No	72 (77.4%)	21 (22.6%)	<0.001*
Dental visit history	Yes	49 (76.6%)	15 (23.4%)	<0.001*
Presumed regular dental checkups	Every six months	46 (52.9%)	17 (24.3%)	<0.001*
Frequency of brushing per day	Three times	55 (87.3%)	8 (12.7%)	<0.001*
Use of toothpaste during brushing	Yes	85 (57.8%)	62 (42.2%)	0.022*
Brushing technique	Circular	0 (0%)	1 (100%)	0.446*
Use of dental floss	Yes	53 (62.4%)	32 (37.6%)	0.041*
Use of mouthwash containing fluoride	Yes	72 (72.7%)	27 (27.3%)	<0.001*
Frequency of consumption sugary food per day	Once	66 (57.4%)	49 (42.6%)	<0.001*
Mouthwash use after sweet consumption	Yes	78 (55.7%)	62 (44.3%)	0.997*
tooth-brushing training received	Yes	87 (56.1%)	68 (43.9%)	0.197*
Mean oral health behavior score (Mean ± SD)	—	6.701 ± 1.373	4.50 ± 1.432	<0.001**

^*^ Fisher’s Exact Test, ^**^ Mann–Whitney U Test

**Table:** Table5. Source of Oral Health Information Among Students Aged 7-9 in Pishva County by ethnicity

Source of Education	Iranian Students (n = 87)	Afghan Students (n = 70)
Parents/Relatives	68 (78.2%)	56 (80.0%)
Television	7 (8.0%)	6 (8.6%)
Others	10 (11.5%)	8 (11.4%)
Don’t know	2 (2.3%)	0 (0%)

**Table:** Table6. Association Between Ethnicity, School Grade Level, and Oral Health Behavior With dmft Index Among Students Aged 7-9 in Pishva County

Variable	Wald Chi-Square	df	Significance Level (P-value)
Intercept	80.192	1	<0.001
School Grade Level	0.179	2	0.914
Ethnicity	70.806	1	0.005
Oral Health Behavior	0.922	1	0.337
Ethnicity * School Grade Level * Oral Health Behavior	22.472	5	<0.001

^*^Generalized Linear Model (GLM); Dependent variable: dmft index.

Among the 157 participants, 87 were Iranian and 70 were from Afghan background. The overall sample comprised 85 boys and 72 girls. Distribution by school grade level showed that 72 participants were first graders, 45 were second graders, and 40 were third graders.

Table-1 Presents the demographic characteristics of the 157 study participants, including gender and school grade level. Of the total sample, 85 participants (54.1%) were male, and 72 (45.9%) were female. Among the 85 male participants, 52 (59.8%) were Iranian, while 33 (47.1%) of the Afghan group were male. In the female group, 35 (40.2%) were Iranian, and 37 (52.9%) were Afghan. No statistically significant difference was observed between the groups in terms of gender (P = 0.078).

Regarding school grade level, a significant difference was observed between the two groups (P = 0.018), as determined by the Chi-square test. Seventy-two participants (45.8%) were in the first grade, while 45 (28.7%) and 40 (25.5%) were in the second and third grades, respectively. In the first grade, 54% (n = 47) of participants were Iranian, while only 35.7% (n = 25) were from the Afghan ethnic group. Among Iranian participants, 25 (28.7%) were in the second grade, and 15 (17.2%) were in the third grade. For Afghan students, 20 (28.6%) were in the second grade, and 25 (35.7%) were in the third grade.

Table-2 Illustrates the distribution of dental health status, as measured by the dmft (decayed, missing, and filled teeth) scores, across different grade levels. Among first-grade students, 20% were categorized as having high dmft scores, 11% exhibited moderate dmft levels, and 9% had very high scores. A smaller proportion of students had low (4%) or very low (1%) dmft values. In the second grade, 16% of students had moderate dmft scores, 10% had high scores, and 3% were classified as very high, while only 1% had low scores and none fell into the very low category. For third-grade students, 14% had moderate dmft levels, 5% had high scores, and 3% had very high dmft values; meanwhile, 3% had low and 1% had very low dmft scores. Overall, higher dmft values were more prevalent in lower grades, particularly among first-grade students.

Table-3 Compares the dental health status (dmft index) between Iranian and Afghan children. Among Iranian children (n = 87), the majority exhibited moderate forty-eight (55%) or high twenty-eight (32%) dmft levels, with only a small proportion classified with very low one (1%) or low seven (8%), and three (3%) with very high dmft scores. In contrast, Afghan children (n = 70) showed a higher prevalence of severe dental decay, with 39 percent (n = 27) falling into the high category and 27 percent (n = 19) into the very high category. Only 3 percent (n = 2) of Afghan children were categorized as very low, and 7 percent (n = 5) as low. Chi-square test was employed to discover statistical relation between these categorical variables with multiple levels. A statistically significant difference was found between the two groups (P < 0.001) based on Chi-Square test. Significantly more Afghan children showed high dmft scores.

Table-4 Shows that Afghan students exhibited significantly lower performance in oral hygiene behaviors compared to their Iranian counterparts (P < 0.001), with a standard deviation of 4.50 ± 1.432. Additionally, the majority of students demonstrated improper tooth brushing techniques, indicating a widespread lack of effective oral hygiene practices. Among Iranian students, 52 (59.8%) and 30 (42.9%) Afghan students reported brushing their teeth to prevent tooth decay. No significant difference was observed between the two ethnic groups in terms of the purpose of tooth brushing (P = 0.026).

Among Iranian children, 22 (73.3%) used soft-bristled toothbrushes, and 47 (80.4%) replaced their toothbrushes every three months. In contrast, only 8 (26.7%) and 11 (19.6%) Afghan students used soft-bristled toothbrushes and replaced their toothbrushes every three months, respectively. A significant difference was observed between Iranian and Afghan children in terms of toothbrush replacement frequency (P < 0.001).

Furthermore, 77.4% (n = 72) of Iranian participants reported no history of toothache, compared to 22.6% (n = 21) of Afghan participants. Toothpaste use during brushing and the habit of dental flossing were reported by 85 (57.8%) and 53 (62.4%) Iranian children, respectively. In comparison, 62 (42.2%) and 32 (37.6%) Afghan children used toothpaste during brushing and flossed their teeth, respectively. Iranian participants also reported more frequent dental visits, with 76.6% (n = 49) attending dental appointments, compared to 23.4% (n = 15) of Afghan participants. Regular dental checkups were considered necessary by 52.9% (n = 46) of Iranian children, while only 24.4% (n = 15) of Afghan children held this belief.

A total of 55 (87.35%) Iranian students brushed their teeth three times a day, while only 8 (12.7%) Afghan students followed the same routine. Usage of fluoride mouthwash and its application after consuming sweet foods was reported by 72.7% (n = 72) and 55.7% (n = 78) of Iranian subjects, respectively. Conversely, 27.3% (n = 27) and 44.3% (n = 62) of Afghan subjects used fluoride mouthwash and applied it after sweet consumption, respectively. In terms of sugary food consumption, 57.4% (n = 66) of Iranian children consumed sugary food once per day, compared to 42.6% (n = 62) of Afghan children.

A significant difference was observed between the two ethnic groups in terms of toothache history, dental visit history, toothbrush replacement frequency, perceived need for regular dental visits, use of fluoride mouthwash, and frequency of sugary food consumption (P < 0.001).

Table-5 Displays that the majority of Iranian (78.2%, n = 68) and Afghan (80%, n = 56) participants obtained their oral health information from parents or other close family members. Additionally, 8% (n = 7) of Iranian and 8.6% (n = 6) of Afghan participants identified television as their primary source of dental information. Furthermore, 11.5% (n = 10) of Iranian and 11.4% (n = 8) of Afghan children reported health centers (public/private) as their main source of oral health information. These findings highlight the need for adequate dental-related information and advice for all children, irrespective of ethnicity.

Table-6 Presents the results of the Generalized Linear Model, which demonstrates a significant effect of ethnicity, as well as a three-way interaction between school grade level, ethnicity, and oral health behavior on the dmft index among students aged 7-9 in Pishva County. The model employed a Poisson distribution with a link function to analyze the relationship between the dependent variable (dmft) and the independent variables. Furthermore, the independent effects of school grade level and oral health behavior were not statistically significant, with P-values of 0.914 and 0.337, respectively.

Model predictors: Ethnicity, School grade Level, Oral Health Behavior, and their three-way interaction.

## Discussion

The present study assessed and compared the dmft (decayed, missing, and filled Teeth) index and oral health behaviors of 7-9-year-old Iranian and Afghan children. To date, no prior studies have specifically addressed the dental health status and behaviors of immigrant populations in Iran. Given the substantial Afghan immigrant population in Iran, Afghan children were chosen for involvement. Based on the Iranian Ministry of Foreign Affairs, in 2020 there were 839,912 Afghan refugees with legal residency cards, 450,000 with temporary passports, and 734,622 undocumented refugees living in Iran [1].

Current evidence suggests that immigrant children and adults often experience greater unmet dental needs and are at an increased risk for oral diseases compared to non-immigrant populations [2]. Contributing factors may include limited financial resources, lack of dental insurance, and reduced access to dental services among immigrant groups [6].

In this study, the findings suggest that Iranian children demonstrated better dental health status and more approving oral health behaviors, including greater awareness of the purpose of toothbrushing, higher frequency of toothbrushing and flossing, lesser frequency of sugar consumption, more regular dental visits, consistent use of fluoridated mouthwash, and better compliance with toothbrush replacement recommendations. However, no significant differences were found between the two groups in terms of the primary source of oral health information, toothbrushing technique use of toothpaste during brushing and purpose of toothbrushing.

Furthermore, Wigen and Wang [16] reported that immigrant children were less consistent in maintaining regular toothbrushing habits compared to their native counterparts. Similarly, the current study found that the dmft score of Iranian children (7.2 ± 4.4) was lower than that of Afghan children (4.5 ± 6.5), indicating a significant correlation between immigration status, socio-cultural factors, and dental health outcomes. Our findings are similar to Gatou et al.’s study [11] in Greece, which found higher caries risk and poorer oral hygiene among immigrant children from low-income areas. The prevalence of untreated caries among first graders in this study (84%) closely mirrors the findings reported by Gatou et al. (84.6%). Similarly, Hoover et al. [17] found that immigrant children in Canada exhibited higher unmet dental needs and greater caries risk compared to native-born Canadian children. Their study reported a higher dmft among immigrant children (5.80 ± 4.24) versus native children (2.4 ± 1.1), a pattern consistent with the results of the present study. Moreover, Nicol et al. [18] reported that immigrant children in Western Australia had a higher mean dmft (6.5 ± 4.5) compared to native Australian children, further emphasizing the role of parental education in influencing children’s oral health outcomes.

Conversely, Alrashidi et al. [19] reported a higher dmft (7.5 ± 2.2) among immigrant children in Texas, USA, compared to the Afghan children examined in the current study. This difference may be attributed to the broader diversity of the immigrant population included in their study, whereas the current study focused solely on Afghan children. Similarly, Al-Ani et al. [20] found that recent immigrant children in Germany had higher dmft scores compared to native German children, corroborating the trends observed in the present study.

It was also found that younger children had higher dmft scores, indicating that age is a contributing factor in the prevalence of dental caries. These findings indicate that younger children, particularly those with less-educated parents, are at a greater risk of developing dental caries—possibly due to their increased dependence on parental care and oral health education [21].

In addition, both group’s source of information was their parents and close families that led to applying incorrect toothbrushing technique. These findings propose an urge for assigning proper information for school children’s parents.

Importantly, this study represents the first effort to assess the dental health status and oral health behaviors of Afghan children residing in Iran. It provides valuable insights into a previously under-researched population and highlights the significance of addressing socio-cultural factors when designing oral health interventions for immigrant groups. The findings suggest that integrating oral hygiene education into school curricula and offering educational courses for immigrant parents at local health centers may serve as effective strategies to improve oral health outcomes within this population.

Although this study offers valuable preliminary insights into the oral health status and behaviors of Afghan immigrant children in Iran, the findings should be interpreted with caution. The study was limited to a single geographic area and did not account for potentially influential confounders, such as household income, access to dental care services, or parental health literacy. Consequently, it cannot be assumed that the observed differences are solely attributable to immigration status or socio-cultural factors. Further research involving larger, more diverse populations and controlling for socioeconomic variables is warranted to better elucidate the complex determinants of oral health disparities among immigrant children.

## Conclusion

In this study, Iranian children aged 7-9 years exhibited lower dmft scores and more favorable oral health behaviors compared to their Afghan peers. While these findings suggest a possible association between immigration status, socio-cultural influences, and dental health outcomes, caution is necessary when interpreting the results. The study’s limited geographic scope and the lack of adjustment for potential confounders, such as socioeconomic status and healthcare accessibility, restrict the generalizability of the conclusions. Future large, multi-center studies that consider these factors are needed to confirm our results and help design better oral health programs for immigrant communities. In addition, as most children obtained their dental health education from their parents and close families, appropriate information should be provided for school children’s parents and distributed through both public and private sectors.

## Conflict of Interest

None declared.
